# Corrigendum to “Stage-specific disruption of X chromosome expression during spermatogenesis in sterile house mouse hybrids”

**DOI:** 10.1093/g3journal/jkac007

**Published:** 2022-02-04

**Authors:** Erica L Larson, Emily E K Kopania, Kelsie E Hunnicutt, Dan Vanderpool, Sara Keeble, Jeffrey M Good

*G3: Genes|Genomes|Genetics*, 2021. https://doi.org/10.1093/g3journal/jkab407

When this paper was first published, there was an error in one of the grant numbers in the funding section. The incorrect text was “E.L.L. was supported by the National Science Foundation (DEB 1557059)”. This should have read “E.L.L. was supported by the National Science Foundation (DEB 2012041)”.

