# Supplementary material for: Stage-specific disruption of X chromosome expression during spermatogenesis in sterile house mouse hybrids
Source: G3 (Bethesda). 2021 Dec 2;12(2):jkab407. doi: 10.1093/g3journal/jkab407 (PMC9210296; doi:10.1093/g3journal/jkab407)
Supplement: jkab407_Supplementary_Figures_Legends [file jkab407_supplementary_figures_legends.pdf]

SUPPLEMENTARY DATA

Table S1: Table of individual male reproductive phenotypes (.csv). Table includes each individual mouse ID (*e.g.* CCPP 21.1M stands for dam x sire, litter number, individual number and sex; CC = CZECHII, PP = PWK, WW = WSB, LL = LEWES), cross type, dates the mice were born and phenotype, their age at phenotyping, measures of body size (weight, body length, tail length, right hind foot, left ear length), weights of paired testes and seminiferous vesicles, counts of motile and nonmotile sperm, counts of total sperm, and counts of sperm head morphology categories.

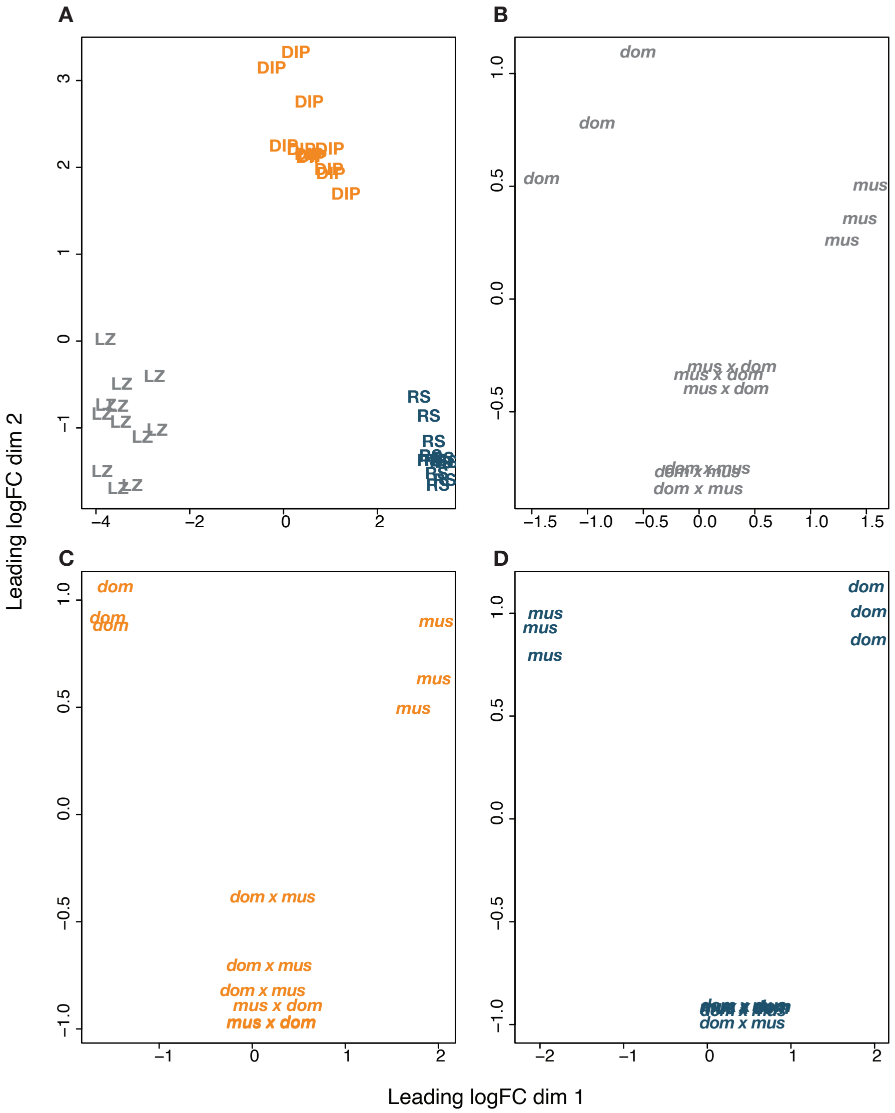

Figure S1. Clustering of gene expression profiles. Multidimensional scaling plots (MDS) of the Euclidean distance among gene expression profiles. Distance approximates the typical log2 fold changes between samples. **A)** RNAseq profiles cluster overall by cell type. LZ = leptotene/zygotene cells (gray), DIP = diplotene cells (orange), RS = round spermatids (blue). **B-D)** Within each cell type, RNAseq profiles cluster by subspecies, with F1 hybrids intermediate to the two parental subspecies. **B)** LZ = leptotene/zygotene cells (gray). **C)** DIP = diplotene cells (orange). **D)** RS = round spermatids (blue)

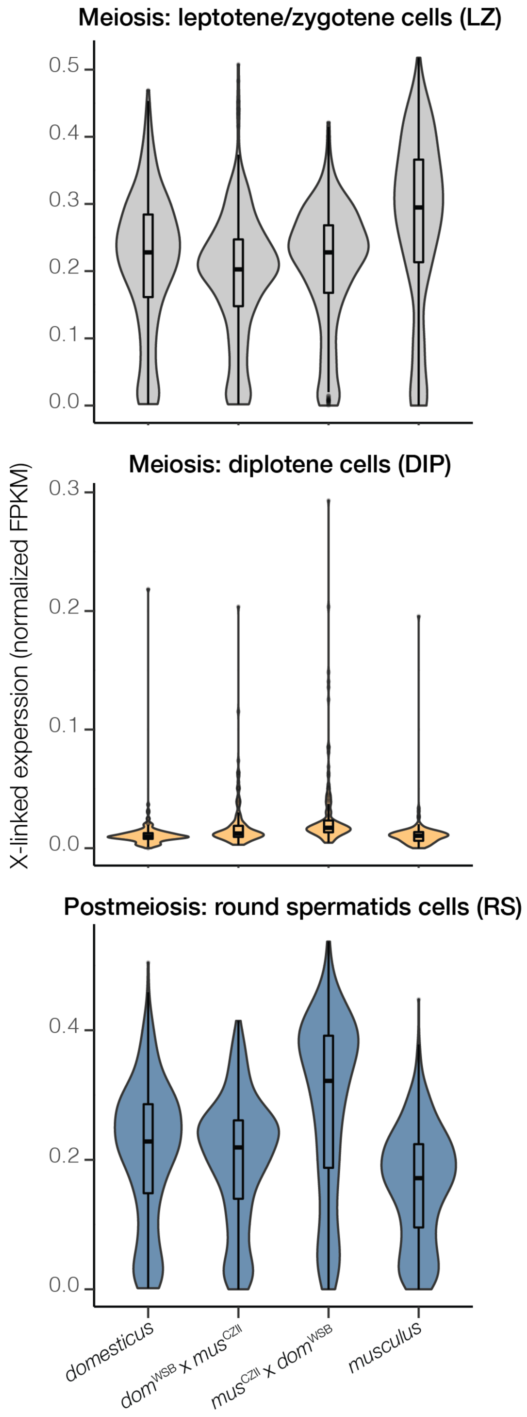

FigS2. X chromosome expression across different cell types and crosses. The distribution of X-linked gene expression in normalized FPKM values (values range 0 to 1). The violin plots show the density of genes with a given expression level and the boxplots depict the median values and quartiles. Gene expression was restricted to genes that have an FPKM > 1 in at least 3 samples per cell type. X-linked expression was elevated in diplotene cells of both hybrids and in round spermatids of ♀ *mus*^{CZII} × ♂ *dom*^{WSB} hybrids.

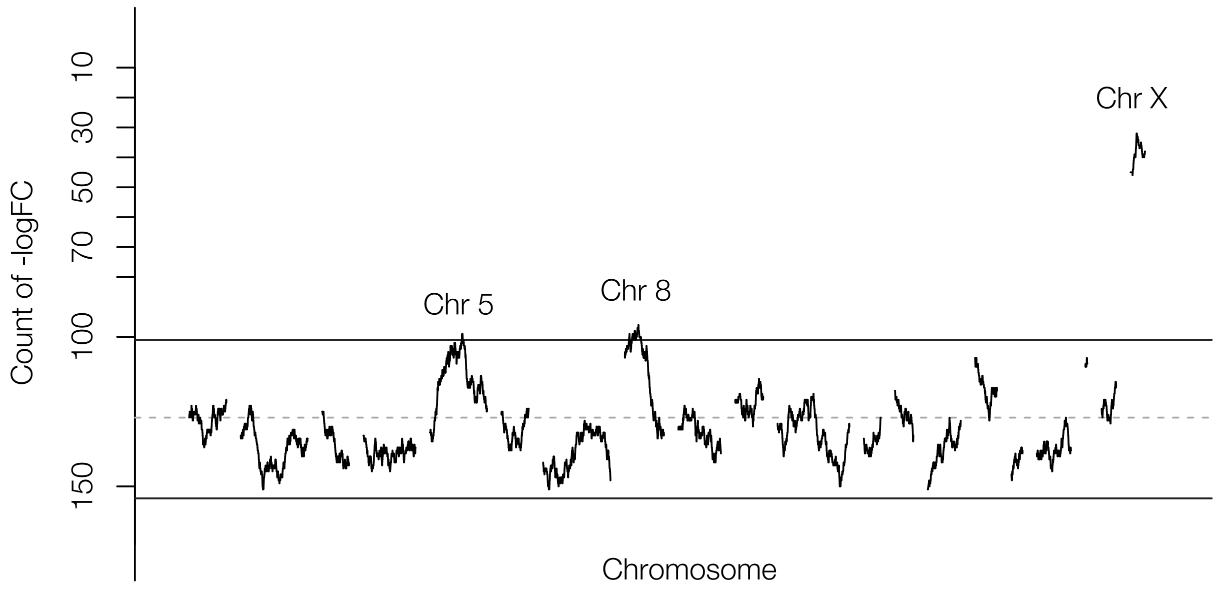

Figure S3. Spatial patterns of postmeiotic expression between subfertile hybrids. Sliding-gene windows (250 genes) for counts of underexpressed genes in postmeiotic cells (round spermatids) between ♀ *mus*^{CZII} × ♂ *dom*^{WSB} hybrids and ♀ *dom*^{WSB} x ♂ *mus*^{CZII} hybrids. Solid lines represent the 99th quantile modeled with a Poisson distribution. Note the Y-axis is plotted so that underexpressed genes fall below the 99th quantile and overexpressed genes are above the 99th quantile. Chromosomes 5 and 8 had relatively small windows of genes overexpressed in *Sly*-deficient ♀ *mus*^{CZII} × ♂ *dom*^{WSB} hybrids, but these windows did not coincide with known multicopy gene families (*Speer*/*α-takusan*).
